# Bridging the gap: large animal models in neurodegenerative research

**DOI:** 10.1007/s00335-017-9687-6

**Published:** 2017-04-04

**Authors:** S. L. Eaton, T. M. Wishart

**Affiliations:** 1 Roslin Institute and Royal (Dick) Veterinary studies, University of Edinburgh, Easter Bush Campus, Edinburgh, EH25 9RG UK; 2 Euan MacDonald Centre for MND Research, Chancellor’s Building, 49 Little France, Edinburgh, EH16 4SB UK

## Abstract

The world health organisation has declared neurological disorders as one of the greatest public health risks in the world today. Yet, despite this growing concern, the mechanisms underpinning many of these conditions are still poorly understood. This may in part be due to the seemingly diverse nature of the initiating insults ranging from genetic (such as the Ataxia’s and Lysosomal storage disorders) through to protein misfolding and aggregation (i.e. Prions), and those of a predominantly unknown aetiology (i.e. Alzheimer’s and Parkinson’s disease). However, efforts to elucidate mechanistic regulation are also likely to be hampered because of the complexity of the human nervous system, the apparent selective regional vulnerability and differential degenerative progression. The key to elucidating these aetiologies is determining the regional molecular cascades, which are occurring from the early through to terminal stages of disease progression. Whilst much molecular data have been captured at the end stage of disease from post-mortem analysis in humans, the very early stages of disease are often conspicuously asymptomatic, and even if they were not, repeated sampling from multiple brain regions of “affected” patients and “controls” is neither ethical nor possible. Model systems therefore become fundamental for elucidating the mechanisms governing these complex neurodegenerative conditions. However, finding a model that precisely mimics the human condition can be challenging and expensive. Whilst cellular and invertebrate models are frequently used in neurodegenerative research and have undoubtedly yielded much useful data, the comparatively simplistic nature of these systems makes insights gained from such a stand alone model limited when it comes to translation. Given the recent advances in gene editing technology, the options for novel model generation in higher order species have opened up new and exciting possibilities for the field. In this review, we therefore explain some of the reasons why larger animal models often appear to give a more robust recapitulation of human neurological disorders and why they may be a critical stepping stone for effective therapeutic translation.

## Why do we need animals to research neurological conditions?

The World Health Organisation specifically “pinpoints neurological disorders as one of the greatest threats to public health” (http://www.who.int) due in part by an ever-increasing ageing population coupled with an exponential increase in the world’s population. There is therefore a critical requirement to improve our knowledge of human neurological disorders which despite their range and prevalence are still remarkably poorly understood. One of the major limitations for advancing our understanding of these conditions is that the majority of data acquired from patient neurological tissue are obtained at the late or terminal stages of disease, where early markers may still be present but are nearly impossible to separate from the late-stage chronic molecular and/or cellular pathology evident in these samples. In order to elucidate the molecular cascades resulting from the neurodegeneration inducing insult in question, and how they alter throughout disease progression, we must examine repeated samples from model systems which are (at least) physiologically representative of three key factors: Firstly, the complexity of the human nervous system; second, the apparent selective regional vulnerability; and third, the differential degenerative progression seen in human neurodegeneration. Currently, we cannot achieve these solely by the use of *in silico* or in vitro studies. There is therefore an urgent demand for reliable animal models that fully mimic neurological disease from genotype to phenotype to regional pathophysiology.

The use of animals for scientific research is an emotive and often a controversial issue. Nonetheless, without animal research, it is likely that the mechanisms underpinning complex neurodegenerative conditions may never be fully understood, new interventions could not be designed and drug therapies could not safely be tested. The justification for using animals in scientific research is widely accepted as a means for improvement of human health either by using models to study diseases or for testing the efficacy of drugs or vaccines (Greek and Menache 2013). What is not widely publicised is the very low rate of translation from drug trial through to successful therapy, with those for neurodegenerative research falling far below the average across the biomedical sciences (Stanzione and Tropepi 2011; Garner 2014). There is certainly room for improvement as the translational statistics (highlighted in detail in Garner 2014) make uninspiring reading for funding bodies and drug discovery companies. Such poor translational figures also give rise to ethical concerns regarding the effective use of animals. To boost both preclinical (animal studies) and clinical (human trials) efficacy at the simplest level, experiments need to have stronger hypotheses (or basis in existing data), be better designed in methodology/technique, be carried out with a much higher degree of accuracy/reproducibility leading to a higher predictive value and reduced attrition (Greek and Menache 2013; Garner 2014). Therefore, an outstanding question remains: what is the best way to make animal studies more accurately translate human neurological conditions?

## Animal models of human diseases

**Fig. 1:**
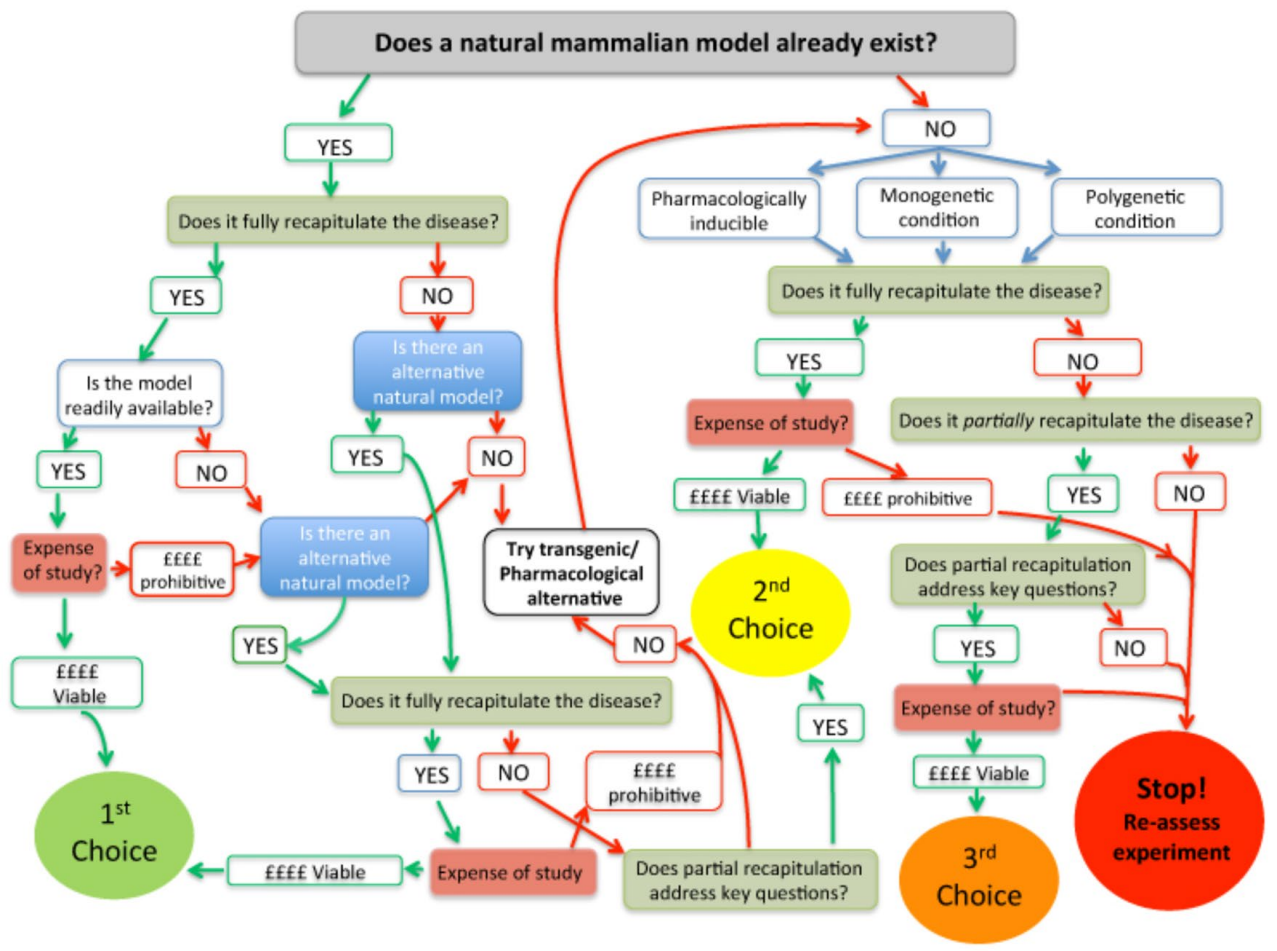
This workflow gives an indication of the many considerations which should be accounted for when selecting the most appropriate animal in which to model a particular disease. The top priority should be to select a model that recapitulates the condition in its entirety; however, these are rare and often the price of carrying out such experiments can be prohibitive. The *green arrows* indicate the positive decisions and possible outcomes, with the *red arrows* representing a pathway which has been rejected and the *blue arrows* indicating alterative models

Model selection is not simple. The criteria for selecting the most appropriate model are complex and broad, ranging from; does this condition already naturally exist in another mammalian system, can a transgenic model be engineered to recapitulate the disease in its entirety, and of course, financial constraints in carrying out pathological time course experiments (see Fig. 1). Funding is extremely competitive and difficult to secure with supporters of biomedical research increasingly focusing on obtaining deliverable targets that are not only reproducible but also translate to direct therapeutic intervention (Bowen and Casadevall 2015). Models then need to be carefully selected in terms of “validity” and “yield” as well as potential for knowledge advancement.

A technique termed reverse translation could be used in the first instance where specific biological markers with significant clinical consequences are first determined in humans and then applied to an animal model to see if they exist and that they reflect the same disease status as the human condition (Garner 2014). Thereafter, novel biomarkers can be identified at different stages of disease in the animal model and ideally they would harbour druggable targets that could delay or even halt the pathological disease process altogether (Garner 2014). For example, the identification and application of biomarkers in human psychiatric conditions which were developed in part using a combination of rodent and non-human primate models have aided the classification of distinct sub-categories of psychoses. These are unbiased and are arguably better diagnostic indicators than purely symptomatic-based analysis (Keifer and Summers 2016).

Reverse translation methods are not always feasible and instead more traditional translational studies are carried out in animal models prior to testing in humans. Biomarkers are still very relevant in these studies, but it is crucial to first understand the mechanistic cascades during the initiating stages of the disease. Thereafter, biomarker identification can occur which may lead to early diagnosis and determination of disease status, as well as strategies for possible therapeutic intervention (Wishart et al. 2012, 2014; Mutsaers et al. 2013).

Disorders that occur naturally in both humans and other animals with similar aetiology have clear benefits. However, it is noteworthy that the predictive validity of any given animal model of neurodegenerative disease will always be impacted upon by physiological and psychological differences that occur between humans and animals (Greek and Menache 2013). Such spontaneous models allow researchers to study degenerative processes and to elucidate and characterise perturbations occurring early in the disease process before symptomatic onset in vivo. This is near impossible to do in human patients. Rodents are the most commonly used mammalian model for generating data from a “large” cohort. They have a relatively short life span, rapid breeding cycles, proven transgenic techniques available (although technolgies such as CRISPR have partially side-lined this as an advantage) and limited space requirements for housing, all of which make them relatively inexpensive to fund and thus an attractive option.

Yet, whilst there are many properties of the rodent models that are advantageous, there are several shortfalls when comparing a rodent to a human including but not limited to size, anatomy and immunological response to infection (Lorenzen et al. 2015; Whitelaw et al. 2016). Furthermore, the relevance of rodent use with regard to certain key features of neurological disorders such as motor impairment and decision making is also questionable due to differences in neuronal network development/maintenance/complexity, reduced white matter content, volume and neuronal density of key targeted regions such as the substantia nigra or complete absence of brain regions containing gyrencephalic cortex, separate caudate and putamen or subthalamic nuclei (Morton and Avanzo 2011; Grow et al. 2016). Such large disparities generally lead to an incomplete recapitulation of a human disorder (see Table 1—detailing the phenotypes of some of the current available models with column eight and nine representing human pathologies which are not recapitulated; Saito et al. (2014), Zahs and Ashe (2015), Philips and Rothstein (2015), Barlow et al. (1996), Jackson et al. (2013), Wang et al. (2016), Bible et al. (2004), Pontikis et al. (2005), Morton et al. (2013), Dawson et al. (2010), Embourg et al. (2007), Yang et al. (2016) and Duque et al. (2015)). It is therefore reasonable to assume that basic biological differences between human and rodent brains may for some cohorts limit our ability to replicate neurodegeneration as observed in patients, especially if those traits are polygenetic in nature (Chang et al. 2015). It is also likely that the evolutionary process may also contribute to interspecies differences observed between models. For instance, it would be reasonable to assume there would be a more robust recapitulation of the human disorder in models with a higher degree of genetic homology to humans. Although Keifer and Summers (2016) argue that core molecular mechanisms may be maintained and share common features throughout vertebrates, is this enough to discover genetic anomalies that are likely to cause subtle or even key differences in the disease process? The fact that rodents have only 48–66% genetic homology with humans, whereas swine and “new world” monkeys have approximately 80% and “old world” monkeys such as the baboon have up to 99%, does beg the question which model is best to fully mimic the condition (Grow et al. 2016; Keifer and Summers 2016). We would therefore propose that neurodegenerative studies in rodents (outside of conditions caused by conserved monogenetic alterations) should be confined to addressing specific questions relating to aspects where the underlying biology is still relevant such as analysis of specific substructures or molecular cascades: For example, analysis of the degenerating spinal cord (Hunter et al. 2016) or beta-catenin and WNT signalling cascades in neurons (Wishart et al. 2014), respectively. Moreover, extrapolation of results from “lower order” mammals should also be limited without adequate evidence of interspecies conservation.

**Table 1:** Human neurodegenerative diseases and models

Disease	Genetic anomaly	Phenotype	Pathology	Model	Natural/engineered		Pathology maintained	Pathology absent	Phenotype	Reference
Alzheimer′s	*APP*	Memory dysfunction	Cerebral cortex atrophy	Rodent	Transgenic	*App* over expression	APP pathology, neuroinflammation	Tau tangles not observed, axonal transport perturbation		Saito et al. (2014)
	*PSEN 1*	Cognitive decline	Extracellular β-amyloid plaques			*Presenilin-1* knock-out	Synaptic defects, cognitive decline, neuronal loss	Perinatal lethality		Zahs and Ashe (2015)
	*PSEN 2*	Dysphagia	Aggregates of hyperphosphorylated Tau protein							
		Dyspraxia	Aberrant processing/clearance of amyloid precursor protein	Canine	Natural		Spontaneous accumulation of Aβ	Tau tangles not observed	Cognitive dysfunction	Holm et al. (2016)
	*APP*			Porcine	Transgenic	hu *App* insertion	No pathology observed	No difference with WT ctrl	Not observed	Dolezalova et al. 2014
Amyotrophic lateral sclerosis (motor neurone disease)	*SOD1* (20% cases)	Muscle atrophy	Degeneration of upper and lower motor neurones	Rodent	Transgenic	*SOD1* knock-in		Cortical motor neuronal degeneration	Age-related muscle atrophy	Philips and Rothstein (2015)
		Dysphagia	Lesion to frontotemporal lobes							
		Dysarthria	Neuroinflammation	Rodent	Natural	Wobbler spontaneous *Vps54*	Reduced number of GABAergic interneurons		Reduced body weight	Moser et al. (2013)
		Spasticity					Marked loss of motor neurons		Head tremor	
									Muscle weakness	
				Porcine	Transgenic	hu *SOD1* knock-in	Nuclear inclusion		Motor defects	Yang et al. (2014)
							Astrogliosis		Muscle atrophy	
Ataxia telangiectasia	*ATM*	Ataxia	Telangiectasias	Rodent	Transgenic	*ATM* knock-out	Neurologic dysfunction	Purkinje cell loss not observed	Low immunity	Barlow et al. (1996)
		Dysarthria	Purkinje cell and granule cell loss						Tumour predisposition	
		Dysphagia		Porcine	Transgenic	SCNT	Purkinje cell loss		Ataxia	Beraldi et al. (2015)
		Increased risk of cancer					cerebellar lesions		Growth retardation	
		Weakened immune system							Motor deficits	
Creutzfeldt–Jakob (CJD)	*PrP*	Ataxia	Accumulation of abnormal form of prion protein	Rodent	Transgenic	hu *PrP* knock-in mutant	Spongiosis, gliosis in hippocampus	Neuronal loss, gliosis in thalamus		Jackson et al. (2013)
		Depression	Vacuolation in grey matter			ovine *PrP* mutants				
		Personality changes	Accumulation of 14-3-3 protein in CSF	Ovine	Natural	Scrapie	Accumulation of abnormal form of prion protein		Ataxia	Houston et al. (2008)
		Memory loss					Vacuolation in grey matter			
Huntington	*HTT*	Hyperkinetic movements	Caudate nucleus atrophy	Rodent *Htt*	Transgenic	*Htt* knock-out	Misfolding of N terminal aggregates	No apoptotic neuronal death		Chang et al. (2015)
		dementia	Loss of GABAergic striatal neurons			*Htt* knock-in	Accumulation of mutant HTT in striatal neurons			
		Psychiatric disturbances	Gliosis							
				Primate	Transgenic		HTT aggregates in the neuronal nuclei	No apoptosis		Wang et al. (2016)
							Degeneration of axons and neuronal processes			
				Ovine	Transgenic		Unknown	Unknown	Circadian rhythm abnormalities	Morton et al. (2013)
				Porcine	Transgenic	Expression of mutant *Htt* fragment	Apoptotic cells		Early death	Yang et al. (2010)
Neuronal ceroid lipofuscinoses (Batten)	*CLN 1–14*	Blindness	Accumulation of ceroid lipofuscin	Rodent	Transgenic	*Ppt1* knock-out	Regional cortical atrophy	Cerebellar atrophy		Bible et al. (2004)
		Cognitive decline	Regional cortical atrophy							
		Motor abnormalities	Neuroinflammation	Rodent	Transgenic	*Cln3* knock-in	Autofluorescent storage material	Regional atrophy		Pontikis et al. (2005)
		Seizures					Progressive neurological deficits			
				Ovine	Natural	*Cln5 & Cln6*	Accumulation of ceroid lipofuscin		Sleep abnormalities	Palmer et al. (2015)
							Regional cortical atrophy		Slow weight gain	
							Neuroinflammation		Postural behavioural changes	
Parkinson′s	*SNCA*	Tremor	Loss of dopaminergic neurons in the substantia nigra	Rodent	Transgenic	*α-synuclein* overexpression	Progressive accumulation of a-synuclein	Fibrillar halo structure of Lewy bodies not observed	Early motor deficits	Yang et al. (2016)
	*MAPT*	Slowness of movement	Loss of dopaminergic projections into striatum				Hyperphosphorylation of NMDA receptor subunit	Cell loss in the substantia nigra not observed		
							Downregulation of glucocerebrosidase			
	*PARK1-16*	Postural instability	Lewy bodies	Rodent	Transgenic	*PINK1* knock-out	Neuronal loss of dopamine/non-dopamine neurons	Lewy body inclusions		Dawson et al. (2010)
	*PINK1*		Accumulation of alpha synuclein inclusions							
	*LRRK2 (PARK8)*			Rodent	Transgenic	*LRRK2* over expression	Neuronal loss of dopamine/non-dopamine neurons	Lewy body inclusions	Motor deficits	Dawson et al. (2010)
				Non-human primate	Injected	*-synuclein* over expression	Loss dopaminergic neurons striatum		Motor deficits	Emborg (2007)
Spinal Muscular Atrophy	*SMN*	Muscle weakness	Lower alpha motor neuron degeneration	Rodent	Transgenic	*Smn* knock-out	Lower alpha neuron degeneration		Muscle weakness	Wishart et al. (2014)
		Paralysis							Paralysis	
				Porcine	Intrathecal delivery of scAAV9		Loss of motor neurons and axons			Duque et al. (2015)

There is therefore a requirement for “more biologically relevant” larger animal models to either bridge small model research with experimental therapeutic trials, or to bypass rodent models that do not readily translate the disease in its entirety.

## Naturally occurring neurological disorders observed in humans and large animals

When trying to model progressive neurological conditions of humans, the once ideal short life cycle of rodents becomes problematic and inbred models cannot truly mimic the diversity of any human population. In contrast, ovine and porcine models become an appealing alternative as they are relatively outbred and are perhaps a more socially acceptable species to use in research compared to close companion animals such as dogs or cats (Pinnapureddy et al. 2015). Interestingly, some disorders already naturally exist in these animals.

### Neuronal ceroid lipofuscinosis

NCLs, commonly known as Batten disease, are a collection of rare lethal inherited neuropathological diseases in children caused by mutations in genes associated with lysosome form and function. Currently, 14 mutations have been discovered and each gives rise to subtly different forms of the disorder, all of which share the majority of pathological and clinical profiles but differing in age of phenotypic onset and duration (Geraets et al. 2016). Shared phenotypic traits include blindness, cognitive and motor impairment, and seizures, and all invariably lead to a greatly reduced lifespan (Geraets et al. 2016).

Several notable discoveries have been determined in a naturally occurring ovine model of Batten disease, including establishing the nature of the so-called “lipofuscin-like” storage material and associating neuroinflammation with regional cortical atrophy (Pinnapureddy et al. 2015). The ovine model closely mimics the human disorder; therefore, any potential molecular targets and therapeutic strategies determined in the sheep could be beneficial across multiple genetic variants in humans (Weber and Pearce 2013; Palmer et al. 2015). Interestingly, a recent study by Amorim et al. (2015) found that many molecular alterations associated with neurodegeneration identified in drosophila and mice models were also conserved in “vulnerable” synaptic compartments in the brain of CLN5 sheep. Such studies reinforce the idea that combined experiments across multiple species, building and refining as we progress, may make for more robust studies for drug targets and subsequent therapies.

Currently, combined lentiviral and Adeno-associated viral (AAV) vector gene therapies have shown some success in the ovine model, and although they are in their infancy, the Batten disease phenotype with combined therapy has not yet been studied (and reported on) at an age when end-stage disease normally occurs (Palmer et al. 2015). So, whilst a “cure” is still a long way off, the utilisation of such large animal models has informed many factors such as gene dosage, delivery routes and viral spread which are extremely important for human therapeutics.

### Prion disease

Scrapie, a naturally occurring disease in sheep, is a prototype for Creutzfeldt–Jakob disease (CJD) in humans with many phenotypic similarities including ataxia, depression and anorexia and sharing typical pathological hallmarks including vacuolation, accumulation of the misfolded protease-resistant form of prion protein as well as neuronal loss (Foster and Hunter 1998). Due to the ability of scrapie to breach the species barrier, researchers have successfully transmitted several strains to more manageable and less financially constraining in-bred mouse models. These models have been used to characterise and elucidate molecular and pathogenic mechanisms that help govern progression of the disease, yet they also have their limitations (Siso et al. 2012).

The ovine model has been instrumental in determining the iatrogenic transmissibility of the disease using blood transfusion studies that would not have been physically possible to carry out in a rodent model. Multiple sampling throughout the time course of this large mammalian model leads to the significant discovery and verification of the theory that not only was whole blood infectious during various stages of the disease but also that various blood fractions harboured infectivity (Yap et al. 1998; Hunter et al. 2002; Houston et al. 2008). The use of large animals in this context for these vital studies thereby alerted public health officials to the fact that iatrogenic transmission was conceivable when receiving a blood or plasma transfusion from a seemingly healthy symptomless donor. In turn, the results of this report have dictated legislation and protocols regarding blood transfusion donation and surgical procedures in the UK (Checchi et al. 2016).

### Duchenne muscular dystrophy

Duchenne muscular dystrophy (DMD) is caused by a mutation in the dystrophin gene with devastating downstream effects that include muscle fibre damage and physical disability and lead to a premature death at around 20 years of age (Rae and O’Malley 2016). This incurable disease affects only male patients giving rise to cognitive impairment with patients exhibiting a lower intelligence quotient (IQ) as well as memory and verbal deficits (Rae and O’Malley 2016). A natural model for DMD occurs in canines and has been well documented around 50 years prior to the development of the currently used genetically engineered mice models. To date, there are approximately 20 canine breeds documented to suffer from DMD, but only a couple of these have been well characterised as experimental models most likely due to them being a companion animal (McGreevy et al. 2015). Canine DMD clinical phenotype and pathology mimic the human disorder almost identically, for example, life expectancy is reduced to 25% and there are extensive and progressive fore limb fibrosis in both species, whilst in rodent models there is only partial recapitulation (McGreevy et al. 2015). Yet despite this, the majority of the research uses rodent models and therapies are yet to be forthcoming as a result (Fuller et al. 2016). There is therefore clear scope to design improved longer term experiments within the DMD field.

### Human immunodeficiency virus

There are a range of neurological disorders associated with human immunodeficiency virus (HIV), which can present with mild clinical signs such as asymptomatic neurocognitive impairment to severe forms of dementia (Elbirt et al. 2015). Often children are more susceptible to cognitive dysfunctions compared to adults infected with HIV (Carryl et al. 2015). Although the incidence of these impairments has diminished since the introduction of combined anti-retroviral therapy, the more mild forms still persist and are associated with an increased risk in age-related degenerative disorders of the central nervous system (CNS) (Elbirt et al. 2015).

Simian immunodeficiency virus (SIV) in monkeys is the prototype for HIV and produces the characteristic neuropathological changes observed in humans infected with HIV (Peeters and Delaporte 2012). A caveat to using SIV-infected monkey models is that only 25% naturally produce encephalitis with drawn out and irregular incubation periods proceeding the development of acquired immune deficiency syndrome (AIDS) (Beck et al. 2015). Although this low proportion of uptake could on the one hand be seen as problematic, researchers could alternatively benefit from elucidating the mechanisms evolved in this natural model to evade transmission of the virus (Chahroudi et al. 2014). However, in order to produce and characterise classic lentiviral encephalitis in an accelerated manner, monkeys can be infected with neurotropic-specific strains of SIV or administered with antibodies that deplete certain immune cell subsets (Beck et al. 2015). With the establishment of lentiviral-induced neurologic SIV, several biomarkers have been detected in blood and cerebral spinal fluid of monkeys, which have been found to correlate with the human condition (Beck et al. 2015; Dezzutti 2015). A positive biomarker status can flag up the greater possibility of a patient developing neurocognitive disorders and clinicians can then treat accordingly, meaning such work has the potential to benefit millions of people worldwide (Elbirt et al. 2015).

## Genetically engineered large animal models

The advent of numerous scientific and technological advances in the twentieth century has resulted in a landmark breakthrough in large animal research, with the successful cloning of Dolly the sheep in turn leading to the cloning of over 20 different species (Pinnapureddy et al. 2015). In the past decade, advancements in gene editor technology have made the production of genetically engineered large animal models more precise and in some cases more “fully” recapitulating human disease (Beraldi et al. 2015; Whitelaw et al. 2016). Whilst technologies such as meganucleases zinc finger nuclease (ZFN), Transcription Activator-Like Effector Nuclease (TALEN), Clustered Regularly Interspaced Palindromic Repeats (CRISPR) and CRISPR associated 9 (Cas9) nuclease are still in their infancy yet have the potential to rapidly create large animal disease models of human neurological conditions to order [discussed in detail by Whitelaw et al. (2016)]. This provides an unprecedented tool for high-quality research and preclinical testing of novel therapeutics.

### Monogenetic disorders

Monogenetic models of disease are technically easier to produce than polygenetic disorders. Yet, despite the simplified genetic underpinnings of such conditions, here we will describe two transgenic large animal models—ataxia telangiectasia (AT) and Huntington′s disease (HD)—demonstrating that even for simple disease-inducing perturbations, they are more successful at recapitulating the neurological disorder in terms of phenotype and pathology than their transgenic rodent counterparts.

#### Ataxia telangiectasia

AT is an inherited multi-systemic disorder caused by mutations in the *ATM* gene. Patients suffer from motor impairment caused by purkinje cell loss, and secondary immune disorders often develop leading to diminished life expectancy of approximately 20–30 years. Beraldi et al. (2015) have produced an excellent large animal model of AT in a pig with homologous mutations of the *ATM* gene using somatic cell nuclear transfer (SCNT) technology. These AT pigs have not only demonstrated characteristic motor impairment but also Purkinje cell loss in the cerebellum, which is a hallmark of the human disease that has never been observed in the rodent models (Beraldi et al. 2015).

It remains unclear why the cerebellum is the target of the *ATM* gene mutation; however, this model has allowed researchers to discover for the first time that Purkinje cell numbers are reduced at birth. This novel finding has not been observed in human patients, due to the nature of sample acquisition at the terminal phase of disease, and therefore further highlights the need to invest in large animal models to try and elucidate the cascades and mechanisms being initiated in the early stage of disease.

#### Huntington′s disease

The cause of Huntington′s disease (HD), an autosomal dominant inherited invariably fatal disorder, is an expansion of a stretch of the huntingtin (HTT) protein (Howland and Munoz-Sanjuan 2014). Pathological aggregation of the mutated HTT protein in striatal, cortical, thalamic and hippocampal neurons as well neuronal cell loss is observed over the time course of this disease. Typical phenotypic hallmarks of HD include severe cognitive deficits, changes in personality as well as hyperkinetic movements of the limbs which are all extremely distressing to the patients and care givers (Ramaswamy et al. 2007).

There are over 25 transgenic rodent models of HD that have been made to date, yet none have accurately mimicked the neurodegeneration seen in humans suffering from this condition (Pouladi et al. 2013; Chang et al. 2015). The normal order of production of transgenic models has meant large animal models of HD have been produced downstream of the rodent models and as a result are still to be fully characterised (Howland and Munoz-Sanjuan 2014). Although in their infancy, there are early indications that non-human primate, ovine and porcine models expressing mutant Htt (small N terminal fragment) are more susceptible to the neurotoxic effects of this expression than rodent transgenics causing early postnatal death, usually within 2–3 days of birth depending on fragment expression (Yang et al. 2010; Li and Li 2012; Chang et al. 2015). Despite their premature death, these models have been found to display phenotypic and pathologic features such as dystonia and apoptotic cells in the brain, which have not been observed in the smaller animal models (Schook et al. 2015; Chang et al. 2015). Although it is still early days, preliminary results from these transgenic large animal models of HD suggest they are more suitable to accurately determine the therapeutic effects of drug delivery agents on HD-specific neurodegeneration.

### Polygenetic disorders

Dementia of advancing age is an ever-increasing problem in the ageing population with huge social and economic costs with Dementia UK estimating an annual financial outlay of £26 billion. There is therefore a matter of urgency to produce robust models to investigate and understand the early mechanisms that lead to the onset of polygenetic disorders such as Alzheimer’s disease (AD) and Parkinson’s disease (PD). However, due to their polygenetic nature and possible environmental influences, it is extremely difficult to engineer genetic models that fully translate these conditions.

#### Alzheimer’s disease

Of the many genes associated closely associated with AD, *APP, PSEN1* and *PSEN2* are the focus of many studies due to their aberrant processing and clearance in the latter stages of AD pathogenesis (Chouraki and Seshadri 2014). Many transgenic mouse models have been produced targeting a single gene mutation, with varied success, but again the short rodent life cycle is problematic for these models as age is one of the biggest risk factors for contracting AD (Hall et al. 2015). With this in mind, pigs became an attractive option for modelling AD disease not only due to the anatomical benefits but also because the identity between human and porcine *APP* is high, with identical secretase cleavage sites and the production of Aβ40 and Aβ42 identical to that produced by humans (Holm et al. 2016). Minipigs expressing *APP695* or *PSEN1* have been engineered using handmade cloning and SCNT technology, but have failed to show any pathological changes 2 and 3 years, respectively, after production (Holm et al. 2016). However, the lack of pathology and behavioural changes may be a direct result of the early time points selected to sacrifice these models. Although researchers aim to unravel pathological events occurring in the preclinical phase of disease, both of these models were euthanised within the first 10% of their lifespan which may be slightly premature to uncover any modifications as most dementia symptoms do not occur usually present until the latter 80% of life (van Vliet et al. 2013; Dolezalova et al. 2014). In contrast, *in vitro* analysis of radial glial cells from mutant *APP* minipigs has detected some early deficits including increased astrogenesis, altered expression of ribosomal and cell cycle genes and increased hyperphosphorylation of Tau (Hall et al. 2015). These could serve as potential indicators into the preclinical aetiology of disease and also add weight to the theory that the minipigs were examined at too early a time point in the incubation period to detect abnormalities consistent with AD phenotypes and pathology. Evolution would suggest that non-human primates should be the most biologically relevant model to study Alzheimer’s disease. There is a high degree of genetic homology (99–100%) of Aβ in great apes and old world monkeys and there are obvious physiological and anatomical similarities (Heuer et al. 2012). Interestingly, despite such biological similarities, there has not been a reported case of AD in NHPs (Toledano et al. 2012). The longevity of NHPs has revealed while they do develop senile plaques and mild forms of cognitive decline such as loss of recognition memory, they have not shown the widespread neuronal loss seen in AD patients (Heuer et al. 2012). This further implies a complex aetiology underpinning AD which is unlikely to be accounted for by aberrations in a single protein.

#### Parkinson’s disease

Age is one of the biggest risk factors for developing Parkinson’s disease (PD) and approximately 1 in 500 people in the UK will develop this progressive neurological condition (Parkinson’s UK). This movement and cognitive disorder have many pathologic hallmarks including formation of intra-neuronal proteinaceous inclusions, Lewy Bodies and the loss of dopaminergic neurons in the Substantia Nigra pars compacta (Jagmag et al. 2015). While there are multiple mutations associated with PD, i.e. alpha-synucleinopathies, there is no single genetic anomaly proven to give rise to PD; instead, there are a range of polygenetic and environmental factors associated with the disease. As a result, many models target either the genetic or toxic induction pathway.

Experimentally induced parkinsonian-like states have been observed in non-human primates, ovine, porcine and feline models by intravenous delivery of the dopamine toxin 1-methyl-4-phenyl-1,2,3,6-tetrahydropyridine (MPTP) (Beale et al. 1989; Frohna et al. 1995; Mikkelsen et al. 1999; Yun et al. 2015). MPTP is lipophilic and crosses the blood–brain barrier easily. To date, it has been the best inducer of Parkinson-like symptomology and pathology in animal models, yet its effect is transient in most mammals with the exception of non-human primates and the Göttingen minipig (Mikkelsen et al. 1999). However, only the primate model has shown expression of Lewy bodies in addition to depletion of dopaminergic neurons in the substantia nigra (Mikkelsen et al. 1999; Yun et al. 2015).

As there are no single gene mutations solely attributed to PD, the many rodent knock-out/in models of single gene edits show variable success at PD recapitulation. There are no successful large animal models with single gene edits that have produced Parkinsonian phenotypes or pathology (Holm et al. 2016). However, a group in China have produced a polygenetic porcine “PD model” with triple gene mutations using CRISPR/Cas9 technology targeting parkin/DJ-1/PINK1 gene loci in pronuclear embryos (Wang et al. 2016). The pigs appear clinically normal at 10 months of age; nevertheless, similarly to the AD minipigs, this would be considered an early point within the incubation period. *In silico* data analysis reported no significant off-target cleavages using this delivery system; therefore, the application of the technology is assumed to have been effective and time will determine the clinical phenotype (Wang et al. 2016). If triple targeted genetic manipulation proves successful, providing comparable data to the human condition, this could potentially bypass the need for rodent models and be a major step forward in future investigations of therapeutics human polygenetic diseases.

#### Amyotrophic lateral sclerosis

The majority of neurological disorders with human correlation that spontaneously develop are in larger mammalian species; however, a naturally occurring murine model of the polygenetic disorder Amyotrophic lateral sclerosis (Holm et al. 2016) exists. The Wobbler mouse has a spontaneous mutation in the *Vps54* gene and exhibits progressive upper and lower motor neuron degeneration, neuronal hyperexcitability and neuroinflammation making it highly comparable to human ALS (Moser et al. 2013). This model has been well characterised in all three phases of the disease (pre-symptomatic, evolutionary and stabilised) since its discovery 60 years ago allowing trials of various compounds including lecithinised superoxide dismutase (Price et al. 1998) (Moser et al. 2013). Following these experiments, a SOD1 transgenic rodent model of ALS was produced in the early 1990s and is currently the most commonly used experimental model of ALS (Park 2015).

Although there are now known to be 21 genes mutations attributed to ALS, the first and most commonly manipulated is still *ALS1* or *SOD1* which accounts for approximately 15% of ALS cases (http://www.alsa.org). To further the understanding of this highly targeted mutation, Yang et al. have produced a transgenic pig that expresses the human SOD1 transgene using the site mutagenesis technique nearly two decades after the production of the first SOD1 transgenic mouse. At the time of publication, the oldest pig was only 2 years of age, yet these pigs exhibited hind limb motor disorder together with motor neuron death, astrogliosis and skeletal muscle atrophy (Yang et al. 2014). Interestingly, this is the only model of ALS to show some nuclear inclusions in motor neurons, located in the intermediate zone and ventral horn of the spinal cord, which also expressed ubiquitin in the nucleus. Considering this is an early time point in the study the larger animal model produces additional information, pathological deficits, which are not observed in the rodent model, and therefore more closely models the human condition.

## Conclusion

In order to better understand disease mechanisms and develop more sophisticated therapeutics, animal models of human conditions are a necessity. This is particularly relevant when considering the complex nature of neurological disorders. We have highlighted many potential limitations of using a rodent to model neurological diseases. These include but are not limited to evolutionary distance/segregation, anatomical heterogeneity, disparate life spans, strain maintenance/inbreeding and physiological anomalies, and can limit our ability to extrapolate to human conditions, especially when considering disorders of a polygenetic nature. Nevertheless, it is important to note that rodent models can and do lead to important discoveries when they are employed to address specific questions.

Appropriate model selection for any disorder but especially those of neurological conditions is a complex process that cannot be underestimated (see Fig. 1). Figure 1 illustrates several possible pathways to decision making of model selection from scientific to economic considerations. With significantly increased competition for funding (perhaps due in part to the lack of successful downstream therapeutics), we have emphasised the importance of this selection process and discussed the need for robust methodological protocols to increase predictive validity. The quality of data derived from these models when taking such considerations into account should therefore lead to more effective use of animals in neurodegenerative research, more physiologically relevant data generation and increased likelihood of translation for therapeutic insights.

Many human neurological disorders naturally exist in large animals making them a useful tool for investigating early/pre-symptomatic events that can be used as biomarkers of disease and/or targets for possible therapeutic interventions. We highlighted the naturally occurring CLN5 ovine model of Batten disease as an excellent example of how a large animal can not only be used in the identification of novel disease determinants, but also in the development of potential therapeutics. However, spontaneous models are relatively limited and there is, and continues to be, a requirement for the production of transgenic models. Until quite recently, construction of transgenic models was only realistic in rodents. However, with the advent of pioneering genetic engineering through sophisticated techniques such as TALEN and CRISPR technologies, the production of large animal transgenic models is now a reality. Although such model production is still in it’s infancy, significant advances have already been made in the AT field with the generation of a porcine model which has already yielded novel insights into embryonic deficits not previously identified.

Ultimately, the key to any successful model of human neurological disease is a robust recapitulation of the condition. Large animal models can often more closely resemble human neurological disorders than their rodent counterparts in terms of both phenotype and pathology, providing an option for bridging the translational gap between rodents and humans.
